# The Viral Load of Human Cytomegalovirus Infection in Children following Hematopoietic Stem Cell Transplant by Chip Digital PCR

**DOI:** 10.1155/2022/2786841

**Published:** 2022-10-17

**Authors:** Wen-Jun Wang, Miao Feng, Feng He, Juan Song, Qin-Qin Song, Dong Xia, Rong Liu, Hai-Lan Yao, Jun Han

**Affiliations:** ^1^State Key Laboratory of Infectious Disease Prevention and Control, National Institute for Viral Disease Control and Prevention, Chinese Center for Disease Control and Prevention, 155 Changbai Rd, Beijing 102206, China; ^2^Department of Biochemistry & Immunology, Capital Institute of Pediatrics, YaBao Rd, Beijing 100020, China; ^3^Department of Hematology, Children's Hospital of Capital Institute of Pediatrics, Beijing 100020, China

## Abstract

**Objective:**

To detect viral load in human cytomegalovirus (HCMV) infection children after hematopoietic stem cell transplant (HSCT) by chip digital PCR (cdPCR).

**Methods:**

The plasmid pUC57-UL83 containing the HCMV-UL83 gene and HCMV AD169 strain were used to evaluate the sensitivity of cdPCR. Either HSV-1, HSV-2, VZV, EBV, HHV-6, or HHV-7 was used to evaluate the specificity of HCMV cdPCR. The cdPCR was compared with quantitative PCR (qPCR) by detecting HCMV infection in 125 children's whole blood samples following HSCT.

**Results:**

The limit of detection (LOD) of HCMV cdPCR was 103 copies/ml and the qPCR LOD was 297 copies/ml for plasmid pUC57-UL83. The result of HCMV cdPCR was 146 copies/ml for the HCMV AD169 strain, indicating that the sensitivity of cdPCR was higher than that of qPCR. There is no cross-reaction between HCMV cdPCR and other herpes viruses. The incidence of HCMV infection was 30.40% in 125 children following HSCT by cdPCR. The range of the HCMV viral load was from 107 copies/ml to 6600 copies/ml by cdPCR.

**Conclusions:**

cdPCR is more sensitive than qPCR for detecting HCMV viral load. Furthermore, the cdPCR could be used to detect the viral load of HCMV infection before or after HSCT in children.

## 1. Introduction

Human cytomegalovirus (HCMV), a ubiquitous *β*-herpesvirus, has been infecting as high as 90% of the human population in developing countries [1]. Most people carry the virus in a latent form. Infection with HCMV could establish a long-lasting immunity to prevent the virus replication from latency to reactivation. The reactivation mostly occurs in immunosuppressed and immunocompromised patients [2–4]. As an important pathogen, HCMV infection causes significant morbidity and mortality in immunosuppressed individuals, especially in patients after hematopoietic stem cell transplant (HSCT) [5–7]. The reason is that the leading target cells of latent infection after primary infection are hematopoietic cells [8–10].

For high-risk children following HSCT, detection of HCMV infection should precede the appearance of clinical symptoms. For the reason that the higher or more rapidly changing viral load would correlate with both the development of HCMV disease and the higher risk of severe HCMV disease in children following HSCT [11–14]. Thus, the quantification of the viral load of HSCT recipients is crucial to the effective patient cure. The most common method is quantitative PCR (qPCR) [15]. However, the qPCR quantification relies on the standard curve, and the diversity of qPCR results in significant variability of the reported quantitative and qualitative data among different laboratories [16–18]. As a result, the divergences in qualitative data could lead to the misjudgment of the development and severity of the disease and the initiation or termination of antiviral therapies.

Our study established a chip digital PCR (cdPCR) method to detect the viral load of HCMV infection before or after HSCT in children and validate its sensitivity, specificity, and repeatability by plasmids and herpes viruses.

## 2. Materials and Methods

### 2.1. Plasmids and Virus

The UL83 gene of HCMV was cloned into the plasmid pUC57 to yield pUC57-UL83. The 3.2 × 10^6^ copies/ml plasmid was a 10-fold serial dilution. The plasmid was double diluted 1 : 2, 1 : 4, 1 : 8, 1 : 16, 1 : 32, 1 : 64 by 3.2 × 10^2^ copies/ml (The copy number of plasmid was obtained by the following formula: (6.02 × 10^23^) × plasmid concentration (g/ml)/(DNA length × 660)). HCMV AD169 strain (5.67 × 10^6^ TCID50/ml) was 10-fold serially diluted from 5.67 × 10^6^ TCID50/ml to 5.67 TCID50/ml. Herpes simplex virus 1 (HSV-1, KOS strain), herpes simplex virus 2 (HSV-2, G strain), varicella zoster virus (VZV, Ellen strain), Epstein–Barr virus (EBV, B95-8 strain), human herpesvirus 6 (HHV-6A, GS strain) (HHV-6 foundation), and human herpesvirus 7 (HHV-7, JI strain) (HHV-6 foundation) stored at −80°C. Each experiment has negative and positive controls. The ddH_2_O was used as the negative control and HCMV AD169 DNA was used as the positive control. The cdPCR and qPCR experiments were repeated three times and these experiments were completed in the second-level biosafety laboratory.

### 2.2. Patients Selection

One hundred twenty-five children following HSCT were enrolled in this study. Male/Female: 73/52, the median age is 7.5 years old. Among 125 children following HSCT, 122 children were allogeneic HSCT, and three were autologous HSCT. And in three autologous HSCTs, both the CMV status (IgG) of the donors (D) and recipient (R) patients prior to transplantation were negative. Among 122 allogeneic HSCT, there were 68 cases of both donor and recipient positive (D^+^ & R^+^), 31 cases of D^+^& recipient negative (R^−^), 18 cases of D^−^ & R^+^, and 2 cases of D^−^ & R^−^. There were 37 cases of acute myelocytic leukemia (AML), 17 cases of acute lymphocytic leukemia (ALL), 22 cases of aplastic anemia (AA), ten cases of myelodysplastic syndrome (MDS), three cases of lymphoma, five cases of neuroblastoma (NB), six cases of Wiskott–Aldrich syndrome (WAS), 25 cases of mucopolysaccharidosis (MPS). HSCT children were collected from May 2018 to May 2020 at the Beijing Capital Institute of Pediatrics Children's Hospital. HCMV viral load in the serum of all recipients was detected by qPCR and cdPCR before HSCT. All children were confirmed according to diagnostic criteria. All subject inclusion was approved by the Ethics Committee of the National Institute for Viral Disease Control and Prevention, China CDC.

### 2.3. Primers and Probe

The primers and probe of cdPCR and qPCR were designed to target the HCMV-UL83 gene.

  HCMV-F 5′-GCAGCCACGGGATCGTACT-3′;
  HCMV-R 5′-GGCTACCTCACACGAGCATT-3′;
  HCMV-Probe 5′-CGCGAGACCGTGGAACTGCG-3′.

### 2.4. qPCR

140 *μ*L diluted viral suspension or clinical whole blood samples were collected, and the DNA was extracted according to the QIAamp Viral DNA Mini Kit (Qiagen, Germany). The final elution volume was 60 *μ*L and stored at −80°C for use. Eight *μ*L DNA templates of each sample were added into a 25 *μ*L qPCR reactions system [19–21], including 12.5 *μ*L of Premix Ex Taq ™ (TaKaRa, Japan), 200 nM of each primer and probe. The cycling procedure is as follows: 95°C for 30 s; 45 cycles of 95°C for 5 s and 60°C for 30 s (CFX96, Bio-Rad, USA).

### 2.5. cdPCR

Eight *μ*L DNA was added into the 25 *μ*L cdPCR reaction system containing 5 *μ*L ToughMix buffer (Stilla, France), 2.5 *μ*L fluorescein (PEXBIO, China), 200 nM of each primer and probe. The cycling procedure is as follows: 95°C, 10 min; followed by 45 cycles of 94°C-5 s and 60°C-30 s. cdPCR was run in Naica™ Crystal Digital PCR system (Stilla, France). Data were analyzed using CrystalMiner.

### 2.6. Statistical Analyses

The data was analyzed by SPSS 20.0 software. Quantitative data were assessed by mean ± standard deviation. Count variables were assessed by the chi-square test. Relations between the expected value of diluted plasmid and those values detected by cdPCR or qPCR were assessed by Spearman's correlation. The linear relation between qPCR and cdPCR was assessed by linear regression. And each result was determined to be significantly different when *P* < 0.05.

## 3. Results

### 3.1. The Sensitivity, Specificity, and Repeatability of cdPCR

The linear dynamic range of the plasmid DNA containing the pUC57-UL83 gene was from 3.2 × 10^6^ to 3.2 × 10 copies/ml. Eight *μ*L of plasmid in each dilution was detected by qPCR and cdPCR. The plasmid with dilution from 3.2 × 10^6^ copies/ml to 3.2 × 10^2^ copies/ml can be detected by qPCR and cdPCR simultaneously. The 32 copies/ml diluted plasmid could not be detected by qPCR and cdPCR. To accurately understand the sensitivity of qPCR or cdPCR, plasmids 3.2 × 10^2^ copies/ml were diluted in a 2-fold series by 1 : 2, 1 : 4, 1 : 8, 1 : 16, 1 : 32, 1 : 64. The limit of detection (LOD) of cdPCR was 103 copies/ml (2.0 copies/reaction) and the LOD of qPCR was 297 copies/ml. The results indicate that the sensitivity of cdPCR was higher than that of qPCR.

**Figure 1:**
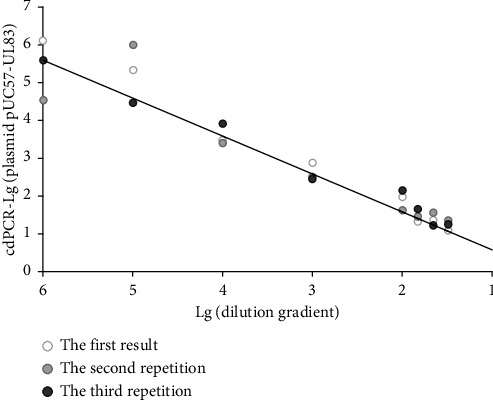
Variations of HCMV DNA copies of cdPCR compared with a standard curve. The black line shows the standard curve of the plasmid DNA. Different scatter points are the HCMV DNA copies tested by cdPCR.

To understand the repeatability of cdPCR, a standard curve of HCMV DNA copy number was established using plasmids. Copies of serially diluted plasmids were detected by cdPCR and the coefficient of variations (CV, standard deviation/mean) of copy number detected by cdPCR was analyzed with a standard curve (Figure 1). The results showed the repeatability of cdPCR was good because the CV value was less than 15%. Good consistency was also observed between the expected value of diluted plasmid and those measured by cdPCR and qPCR (*R* = 0.979, *P* < 0.05 for cdPCR and the expected value of diluted plasmid, *R* = 0.939, *P* < 0.05, for qPCR and the expected value of diluted plasmid).

To determine the specificity of cdPCR for HCMV, 7 herpes viruses, including HSV-1, HSV-2, VZV, EBV, HCMV, HHV-6A, and HHV-7, were tested by cdPCR, respectively. The results showed that cdPCR only detected the HCMV AD169 strain but not the other 6 herpes viruses, suggesting that cdPCR for HCMV has no cross-reaction with other herpes viruses.

### 3.2. Validation of cdPCR Using HCMV AD169 Strain

Eight *μ*L HCMV DNA each dilution by 10-fold dilution from 5.67 × 10^6^ TCID50/ml to 5.67 TCID50/ml was tested by qPCR and cdPCR, respectively. HCMV DNA from 5.67 × 10^6^ TCID50/ml to 5.67 × 10 TCID50/ml virus could be detected by qPCR but not for 5.67 TCID50/ml. However, HCMV DNA of 5.67 TCID50/ml virus could be detected by cdPCR, which was 146 copies/ml (2.7 copies/reaction). The results showed that the sensitivity of cdPCR was better than that of qPCR.

### 3.3. HCMV Infection of Children following HSCT

To verify the sensitivity of the cdPCR method and whether it can be used for HCMV detection in the blood of HSCT patients, 125 children's whole blood samples following HSCT were tested by qPCR and cdPCR. Thirty-four samples were positive through qPCR and cdPCR, ninety-one samples were negative through qPCR. However, 4 out of 91 qPCR negative samples were positive by cdPCR. The results showed that the method of cdPCR was more sensitive than qPCR for HCMV detection (Table 1).

Subsequently, one hundred twenty-five children's whole blood samples following HSCT were tested by cdPCR to investigate the viral load of HCMV infection. The HCMV viral load was from 107 copies/ml to 6600 copies/ml by cdPCR. In 68 cases of D^+^ & R^+^, HCMV was detected in 28 cases (41.18%), and the viral load was 107–6600 copies/ml. HCMV was detected in 7 cases (22.58%) with a viral load of 437 to 5314 copies/ml in 31 D^+^ & R^−^ cases. There were 18 cases of D^−^ & R^+^, and three cases (16.67%) of HCMV were detected, and the viral load was 463–883 copies/ml. There were 5 cases of D^−^ & R^−^, and HCMV was not detected. The HCMV infection rate was 40.54% (15/37) among AML cases, and the HCMV viral load was from 107 copies/ml to 5314 copies/ml. The HCMV infection rate was 41.18% (7/17) in ALL cases, and the HCMV viral load was from 137 copies/ml to 1779 copies/ml. The HCMV infection rate was 22.73% (5/22) among AA cases, and the HCMV viral load was from 154 copies/ml to 6600 copies/ml. The HCMV infection rate was 40% (4/10) among MDS cases, and the HCMV viral load was from 999 copies/ml to 5957 copies/ml. The HCMV infection rate was 33.33% (1/3) among lymphoma cases, and the HCMV viral load was 2494 copies/ml. There was no HCMV infection in NB cases. One case (16.67%) was infected with HCMV in WAS cases, and the HCMV viral load was 801 copies/ml. The HCMV infection rate was 20% (5/25) among MPS cases, and the HCMV viral load was from 351 copies/ml to 5100 copies/ml (Table 2). Due to the small number of cases, the HCMV infection rate of patients with different primary diseases is not statistically different, and the viral load of HCMV infection varies among different diseases group without significant variation.

The detection rate of HCMV was 30.40% (38/125) in 125 children following HSCT, and the range of HCMV viral load was from 107 copies/ml to 6600 copies/ml. The detection rate in the male group was 30.14% (22/73) and in the female group was 30.77% (16/52). The detection rate of HCMV was 89.47% (34/38) in the HCMV-positive children following HSCT aged 0–12. In the aged 0–6 group, the detection rate in males was 25.64% (10/39) and in females was 22.58% (7/31). In the aged 7–12 group, the detection rate in males was 39.29% (11/28) and in females was 40% (6/15). For over 12 years old children following HSCT, the HCMV detection rate was 33.33% (4/12), the detection rate in males was16.67% (1/6), and the detection rate in females was 50% (3/6) (Table 3), suggesting that HCMV infection is mainly found in patients under 12 years of age following HSCT, and the gender does not affect HCMV infection rate.

The prognosis of 125 children following HSCT was analyzed retrospectively. GVHD was found in 13 children (the range of the HCMV viral load was from 437 copies/ml to 4457 copies/ml, and the median of viral load was 693 copies/ml) (34.21%) of 38 HCMV-positive children and 25 children (28.74%) of the 87 HCMV-negative children (*P* > 0.05). HCMV-positive children were slightly more likely to develop GVHD than HCMV-negative children. Among the 38 positive children, five children died following HSCT. The coinfection with EBV occurred in 2 of the 5 deaths.

## 4. Discussion

The increase of HCMV viral load in clinical samples can predict disease progression and outcome in patients [11]. However, the lack of well-established viral load thresholds has limited HCMV qPCR in clinical applications. The results of qPCR cannot be directly compared between different hospitals without consensus standardization. Thus, it is hard to use the viral loads' value to initiate preemptive therapy for patients infected with HCMV [14, 16, 22]. A variety of factors can lead to large changes in viral load, such as detection methods, disease severity (course), and sample quality [23–25].

Digital PCR solves the shortcomings of qPCR. Droplet digital PCR (ddPCR) and cdPCR are two types of commercial digital PCR technic. As our results, many studies show that the sensitivity of digital PCR is significantly higher than that of qPCR [20, 26, 27]. Furthermore, the sensitivity of ddPCR for HCMV is 100 copies/ml [28]. Our results also showed that the lowest viral load of cdPCR in detecting whole blood samples was 107 copies/ml, lower than qPCR. In the clinical context, qPCR is a common method for detecting HCMV viral load. But the sensitivity of qPCR was less than that of cdPCR. The cdPCR can detect a lower viral load of HCMV infection than qPCR under its threshold. The cdPCR is conducted through an advanced cutting-edge microfluidic chip (Sapphire chip) 2D array of microchamber to complete PCR reaction, and cdPCR can conduct three-color multiplexing amplification [29, 30]. Due to simplified steps, cdPCR effectively reduces the risk of contamination. Thus, in this study, an HCMV cdPCR method was established to detect the viral load of HCMV infection.

It is well known that HCMV is the most common transmissible virus in children following HSCT and is considered to be the major risk factor for transplantation. Studies have shown that almost all HCMV viremia after bone marrow transplantation occurs in HCMV-positive recipients, and only a few patients can be transmitted from the donor [31]. For patients following HSCT, myeloablation may reduce immunity and lead to HCMV reinfection [32]. Our results also showed that the HCMV detection rate was 57.85% in HCMV-positive recipients prior to transplantation, which was higher than that in HCMV-negative recipients prior to transplantation (22.58%). Unlike those who have undergone solid organ transplantation, the HCMV-positive donor is the high-risk group [32].

In this study, up to 30.40% of samples were positive for HCMV by cdPCR. About 90% of HCMV infections occurred before the age of 12 in these children following HSCT. Among 125 children following HSCT, neoplastic diseases (AML, ALL, and lymphoma) have a higher HCMV infection rate than non-neoplastic diseases (AA, WAS, and MPS). It is reasonable that the immune status of neoplastic children is weaker than that of non-neoplastic children, which makes HCMV infection more likely to occur. Our results suggest that HCMV-positive children were more likely to develop GVHD than HCMV-negative children, consistent with previous studies. In other words, it is necessary to pay attention to children following HSCT with HCMV and HCMV-infected children after bone marrow transplant. Therefore, timely monitoring and accurate quantification of HCMV viral loads in children following HSCT can provide an important basis for clinical antiviral treatment.

## 5. Conclusion

In conclusion, the cdPCR method of HCMV DNA was established and confirmed for detecting HCMV viral load in this study.

## Figures and Tables

**Table 1:** Comparisons between qPCR and cdPCR.

qPCR	cdPCR		Total	*P*
	Positive	Negative		
Positive	34	0	34	<0.05
Negative	4	87	91	
Total	38	87	125	

**Table 2:** Characteristics of 125 children following HSCT.

Children patient characteristics		No. of children patients (no. of HCMV Positive)
Age (years)	0–6	70 (17)
	7–12	43 (17)
	≥12	12 (4)

Sex	Male	73 (22)
	Female	52 (16)

HCMV IgG	D^+^ & R^+^	68 (28)
	D^+^ & R^−^	31 (7)
	D^−^ & R^+^	18 (3)
	D^−^ & R^−^	5 (0)

Disease	AML	37 (15)
	ALL	17 (7)
	AA	22 (5)
	MDS	10 (4)
	Lymphoma	3 (1)
	NB	5 (0)
	WAS	6 (1)
	MPS	25 (5)

HSCT	Allogeneic	122 (38)
	Autologous	3 (0)

Source of HSCT	BM + PBSCT	98 (32)
	PBSCT	18 (3)
	UCB-HSCT	9 (3)

GVHD (grade)	0	83 (23)
	1-2	30 (12)
	3-4	12 (3)

Abbreviations: HSCT, hematopoietic stem cell transplantation; HCMV, human cytomegalovirus; D, donor patients; R, recipient patients; AML, acute myelocytic leukemia; ALL, acute lymphocytic leukemia; AA, aplastic anemia; MDS, myelodysplastic syndrome; NB, neuroblastoma; WAS, Wiskott–Aldrich syndrome; MPS, mucopolysaccharidosis; BM, bone marrow; PBHSCT, peripheral blood stem cell transplantation; GVHD, graft vs. host disease.

**Table 3:** HCMV infection rate by cdPCR in children following HSCT.

Age	No.	HCMV positive	Positive rate (%)	Male			Female		
				Positive	Negative	Positive rate (%)	Positive	Negative	Positive rate (%)
0–6	70	17	24.28	10	29	25.64	7	24	22.58
7–12	43	17	39.53	11	17	39.29	6	9	40
≥12	12	4	33.33	1	5	16.67	3	3	50
Total	125	38	30.40	22	51	30.14	16	36	30.77

## Data Availability

The data used to support the findings of this study are available from the corresponding author upon request.
